# Reply to De Marco et al.

**DOI:** 10.1016/j.advnut.2025.100410

**Published:** 2025-04-15

**Authors:** Dan Liang, Li Wang, Zhen Luo, Changwen Ke, Yingsi Lai

**Affiliations:** 1 From the Department of Immunology and Microbiology, College of Life Science and Technology, Jinan University, Guangzhou, China; 2 Department of Medical Statistics, School of Public Health, Sun Yat-sen University, Guangzhou, China; 3 International Institute for Translational Chinese Medicine, School of Chinese Materia Medica, Guangzhou University of Chinese Medicine, Guangzhou, China; 4 BSL-3 Laboratory (Guangdong), Guangdong Provincial Key Laboratory of Tropical Disease Research, School of Public Health, Southern Medical University, Guangzhou, China; 5 Guangdong Provincial Key Laboratory for Emergency Detection and Research on Pathogen of Emerging Infectious Disease, Guangdong Provincial Center for Disease Control and Prevention, Guangdong Workstation for Emerging Infectious Disease Control and Prevention, Guangzhou, Guangdong, China

Dear Editor:

We appreciate the thoughtful commentary by De Maio et al. regarding our recent study on global iodine deficiency trends [1]. Their insights into population homogeneity and salt iodization effects provide valuable context for interpreting public health interventions. We would like to address 3 key points raised:

1. Homogeneous populations as natural experiments

We concur that isolated communities with stable genetic/environmental profiles (e.g. Southern Italy cohort) offer unique opportunities to isolate iodine intervention effects. Our model currently incorporates regional socioeconomic covariates (Human development index (HDI)), but genetic homogeneity parameters could enhance predictive accuracy. Future iterations may include population stratification indices from genomic databases.

2. Thyroid nodule epidemiology

The reported 46% reduction (86%→40%) in thyroid nodules postiodization aligns with WHO estimates of 30%–50% goiter prevalence decline in iodine-sufficient populations [2]. However, ultrasonography sensitivity improvements since 1993 may partially confound these observations. We encourage the authors to publish full methodology for comparative analysis with our global ultrasonography-adjusted data set.

3. Migration and globalization

This critical factor is currently modeled through dynamic socioeconomic covariates and WHO migration-adjusted urinary iodine concentration metrics [3]. We propose collaborating to integrate the authors’ microlevel migration/nutrition data into our macrolevel model, particularly for Mediterranean regions.

We thank De Maio et al. for highlighting the importance of real-world laboratory populations. Their findings reinforce the need for layered modeling approaches that reconcile localized biological signals with global health trends.

## Conflict of interest

The authors report no conflicts of interest.
